# Acute kidney injury in children hospitalized for community acquired pneumonia

**DOI:** 10.1007/s00467-021-05022-x

**Published:** 2021-03-20

**Authors:** Pierluigi Marzuillo, Vincenza Pezzella, Stefano Guarino, Anna Di Sessa, Maria Baldascino, Cesare Polito, Emanuele Miraglia del Giudice, Felice Nunziata

**Affiliations:** 1 Department of Woman, Child and of General and Specialized Surgery, Università della Campania “Luigi Vanvitelli”, Via Luigi De Crecchio 2, 80138 Napoli, Italy; 2 Department of Pediatrics, AORN Sant’Anna e San Sebastiano, Via Ferdinando Palasciano, 81100 Caserta, Italy

**Keywords:** Acute kidney injury, Community acquired pneumonia, C-reactive protein, Children

## Abstract

**Background:**

Acute kidney injury (AKI) enhances the risk of later chronic kidney disease. Significant prevalence of AKI is reported in adults with community acquired pneumonia (CAP). We investigated prevalence of and prognostic factors for AKI in children hospitalized for CAP.

**Methods:**

We retrospectively collected clinical and biochemical data of 186 children (48.4% male; mean age 2.6±2.4 years) hospitalized for X-ray-confirmed CAP. AKI was defined according to Kidney Disease/Improving Global Outcomes creatinine criteria. We considered as basal serum creatinine the value estimated with Hoste (age) equation assuming basal eGFR were median age-based eGFR normative values for children ≤ 2 years of age and eGFR= 120 mL/min/1.73m^2^ for children > 2 years. Univariate and multivariate logistic regression models were used to explore associations with AKI.

**Results:**

AKI was found in 38/186 (20.4%) patients. No patient required hemodialysis nor reached AKI stage 3, 5 (2.7%) reached AKI stage 2, and 33 (17.7%) AKI stage 1. Mean length of stay was 6.0±1.7, 6.9±2.3, and 12.2±1.5 days, for patients without AKI, stage 1 AKI, and stage 2 AKI (*p* < 0.001), respectively. Duration of symptoms before hospitalization (OR 1.2; 95%CI 1.09–1.43; *p* = 0.001), severe pneumonia (OR 11.9; 95%CI 4.3–33.3; *p* < 0.001), and serum C-reactive protein levels (OR 1.1; 95%CI 1.04–1.23; *p* = 0.004) were independent AKI predictors.

**Conclusions:**

About 1/5 of children hospitalized for CAP present a generally mild AKI with a longer stay for those with more severe AKI. Attention should be paid to kidney health of children with CAP especially in presence of higher duration of symptoms before hospitalization, severe pneumonia and higher serum CRP levels.

**Graphical Abstract:**

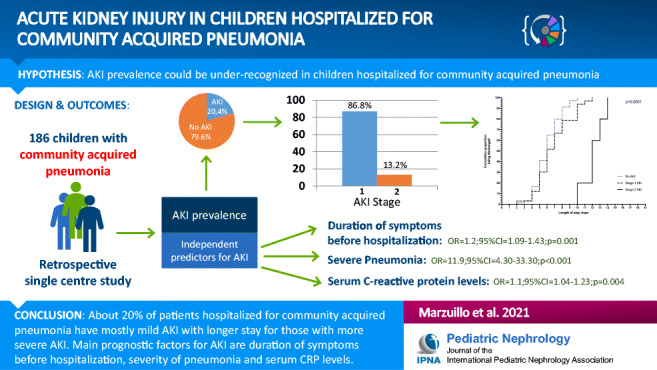

**Supplementary Information:**

The online version contains supplementary material available at 10.1007/s00467-021-05022-x.

## Introduction

Childhood community acquired pneumonia (CAP) accounts for approximately 2 million outpatients visits annually and is among the most common causes for pediatric hospitalization annually in the United States [1]. Adults with CAP present a considerable risk to develop both acute kidney injury (AKI) [2–5] and later chronic kidney disease (CKD) [6].

We hypothesized that AKI prevalence could be under-recognized in children hospitalized for CAP especially for milder forms. Even a milder form of AKI, however, doubles the risk of CKD [7], and from this view, it is important to detect any AKI episode in order to plan a proper follow-up for the patients having shown an AKI.

Because no previous studies have investigated the relationship between CAP and AKI in childhood, we aimed to investigate the prevalence of and prognostic factors for AKI in children hospitalized for CAP.

## Methods

We retrospectively collected the data of all patients consecutively discharged with CAP diagnosis from the Pediatric Department of Sant’Anna e San Sebastiano Hospital, Caserta, Italy, from January 1, 2017, to December 31, 2019. This pediatric ward is placed in a general hospital especially devoted to care of adults.

**Fig. 1:**
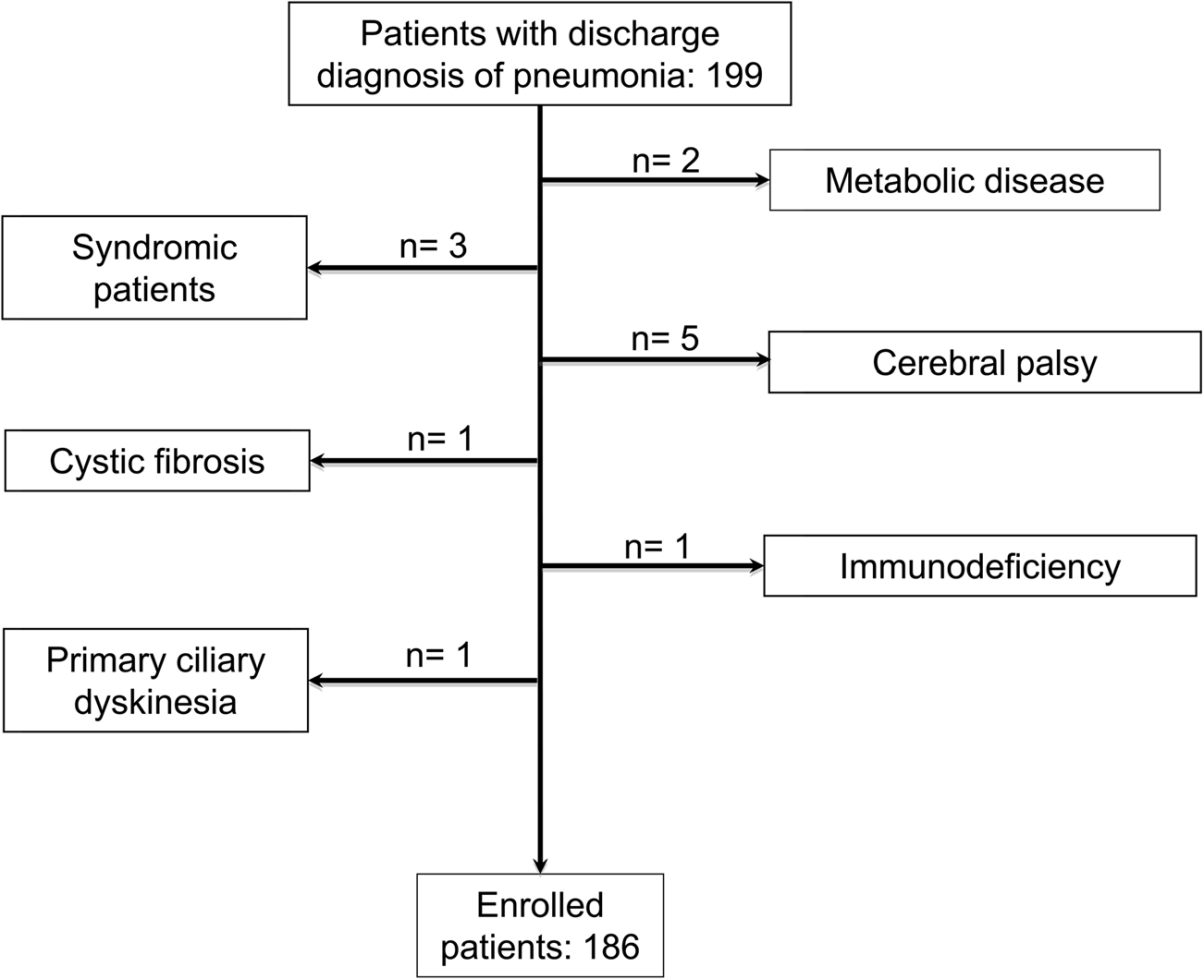
Flow-chart describing patient enrolment

Inclusion criteria were age < 18 years, discharge primary diagnosis of X-ray-confirmed CAP, and availability of serum creatinine levels at admission. We excluded patients with pre-existent comorbidities potentially affecting the severity of pneumonia (Figure 1). Serum creatinine levels at admission were available for all patients with CAP evaluated during the study period. Our Research Ethical Committee approved the study (approval n° 12770/2020) and informed consent was obtained before any procedure.

### Data collection

From the digitalized clinical charts, we collected the clinical and laboratory characteristics listed in the Table 1 as well as history of known nephro-urological disease. We recorded all serum creatinine values obtained during hospitalization. In 131 out of 186 (70.4%) patients, we had at least one other serum creatinine determination in addition to that at admission. Among these 131 patients, all the patients with AKI were included. Serum creatinine was determined in 69 out of 131 patients after 48 h of stay (52.7%), in 40 (30.5%) after 72 h, and in 22 (16.8%) after 96–240 h.

**Table 1:** Clinical and laboratory characteristics of children hospitalized for CAP with and without AKI

	All patients No.= 186	AKI (no) No.= 148	AKI (yes) No.= 38	p
Age, yr	2.6±2.4	2.6±1.9	2.9±2.4	0.35
Male gender, No. (%)	90 (48.4)	76 (51.3)	14 (36.8)	0.10
Birth weight°, kg	3.2±0.6	3.25±0.5	3.0±0.8	0.037
Preterm birth°, No. (%)	29 (15.6)	19 (12.8)	10 (26.3)	0.04
Duration of symptoms before admission, days	4.6±3.8	4.1±3.0	6.7±5.3	<0.001
Weight, percentiles	51.5±31.9	52.0±32.0	49.6±32.1	0.67
Signs of respiratory distress, No. (%)	50 (26.9)	34 (23.0)	16 (42.1)	0.017
Respiratory rate >2SDS*, No. (%)	35 (36.5)	25 (31.6)	10 (58.8)	0.038
Oxygen saturation at admission, %	96.8±2.2	96.1±2.4	95.9±3.1	0.70
Need of oxygen therapy, No. (%)	20 (10.8)	11 (7.4)	9 (23.7)	0.0039
Highest FiO2, %	30.7±6.6	29.8±8.4	31.8±3.9	0.52
Duration of oxygen therapy, days	2.2±1.0	1.6±0.8	3.2±0.4	0.001
Need of high flow oxygen therapy, No. (%)	1 (0.5)	0 (0)	1 (2.6)	0.047
Need of intubation, No. (%)	1 (0.5)	0 (0)	1 (2.6)	0.047
Use of nephrotoxic drugs+, No. (%)	25 (13.4)	18 (12.2)	7 (18.4)	0.30
Need of beta2-agonists, No. (%)	94 (50.5)	76 (51.3)	18 (47.4)	0.66
Use of systemic steroids, No. (%)	72 (38.7)	60 (40.5)	12 (31.6)	0.31
Presence of fever, No. (%)	98 (52.7)	70 (43.7)	28 (73.7)	0.004
Maximal body temperature, °C	38.2±1.1	38.1±1.1	38.5±0.8	0.05
Refill >2 seconds, No. (%)	4 (2.1)	1 (0.7)	3 (7.9)	0.006
HR>2SDS for age, No. (%)	45 (24.2)	33 (22.3)	12 (31.6)	0.86
Severe pneumonia, No. (%)	37 (19.9)	17 (11.5)	20 (52.6)	<0.001
Glasgow Coma Scale <15, No. (%)	3 (1.6)	2 (1.3)	1 (2.6)	0.57
Need of intravenous rehydration, No. (%)	61 (32.8)	49 (33.1)	12 (31.6)	0.86
Need of bolus, No. (%)	4 (2.1)	1 (0.7)	3 (7.9)	0.006
WBC, n/mcL	13703.1±7974.7	13838.0±8095.3	13177.7±7567.0	0.65
Neutrophils, n/mcL	8481.8±4881.2	8236.2±4959.8	9431.8±4500.1	0.18
Serum creatinine levels at admission, mg/dL	0.34±0.14	0.3±0.1	0.5±0.15	<0.001
eGFR at admission, mL/min/1.73m^2^	111.9±52.1	122.3±52.8	71.6±19.4	<0.001
Serum urea levels, mg/dL	10.4±3.6	10.0±3.3	11.8±4.0	0.005
Hematocrit >2SDS, No. (%)	16 (8.6)	15 (10.2)	1 (2.6)	0.14
Hematocrit <2SDS, No. (%)	59 (31.7)	37 (25.0)	22 (42.1)	0.037
C-reactive protein, mg/dL	3.5 (4.9)	4.0 (4.8)	8.2 (8.4)	<0.001
Length of stay, days	6.3±2.0	6.0±1.7	7.5±2.9	<0.001

^*^This data was available for 96 patients, 79 without AKI and 17 with AKI

^+^Twenty-four patients assumed ibuprofen and 1 intravenous aminoglycosides

°There was no difference in age at admission comparing patients with prematurity/low birth weight (median 2.08, interquartile range 2.5) compared with the others (median 2.08, interquartile range 2.41) (*p* =0.67)

For normally distributed variables means ± SDS are shown, while for non-parametric ones median and interquartile range are shown

*Abbreviations:* *AKI* acute kidney injury, *CAP* community acquired pneumonia, *eGFR* estimated glomerular filtration rate, *FiO2* Fraction of inspired oxygen, *HR* heart rate, *SDS* standard deviation score, *WBC* white blood cell count

### Case definition

Serum creatinine concentration was measured by IDMS-traceable method.

AKI was defined by the Kidney Disease/Improving Global Outcomes (KDIGO) serum creatinine criteria [8]. The basal serum creatinine value was estimated using previously validated back-calculation methods [9]. As the height was missing in some clinical charts, and as height-dependent and height-independent basal serum creatinine estimation methods were comparable [9], we calculated the estimated glomerular filtration rate (eGFR) by Hoste (age) equation [10]. This equation was also used to back-calculate basal serum creatinine assuming that basal eGFR were the median age-based eGFR normative values for the children ≤ 2 years of age [11] and eGFR = 120 mL/min/1.73 m^2^ for children > 2 years [12].

No AKI, stage 1, stage 2, and stage 3 AKI were defined by creatinine values < 1.5, 1.5 to < 2, 2 to < 3 and ≥ 3 times the basal creatinine, respectively [8].

We did not consider the KDIGO urine output criteria because the urinary output measurement was lacking.

### Other definitions

CAP was defined as the presence of signs and symptoms of pneumonia in a previously healthy child due to an infection which has been acquired outside hospital [13].

The severity of CAP was evaluated according to the World Health Organization definition [14]. In brief, the pneumonia was retrospectively classified as severe if the patients presented any one of oxygen saturation < 90%, central cyanosis, severe respiratory distress, inability to drink or breastfeed or vomiting, altered consciousness, and convulsions [14].

On the basis of the chest X-ray findings, the pneumonia was defined as lobar in case of a non-segmental, homogeneous consolidation predominantly involving one lobe with air bronchograms [15]. Bronchopneumonia was defined by inhomogeneous patchy areas of consolidation involving one or more lobes, while interstitial pneumonia was defined by reticular or reticulo-nodular pattern [15]. All radiographs were blindly reviewed by the same operator.

The heart rate (HR) and respiratory rate (RR) were compared with percentiles for age and body temperature [16, 17]. Increased HR and RR were defined by values > 2 standard deviation score (SDS). Impairment of consciousness was defined as present or absent. We defined the impairment of consciousness as present in case of Glasgow coma scale < 15. Hematocrit less or more than 2SDS was evaluated on the basis of age-specific percentiles [18]. Fever was defined by body temperature > 38.5 °C. Birth was considered as preterm when babies were born before 37 weeks of pregnancy had been completed [19].

### Post-hoc power calculation

No previous studies have investigated the AKI prevalence in children with CAP. Studies in adults reported AKI prevalence in CAP ranging from 4.3 to 34% [2–5]. On the basis of the median AKI prevalence of 13.1% in adults [2–5], considering a prevalence of AKI of 20.4% in our population of 186 subjects, the calculated post hoc power, with an alpha of 0.05, was 80.8%.

### Statistical analysis

P values < 0.05 were considered statistically significant. Differences for continuous variables were analyzed with the independent-sample *t* test for normally distributed variables and with the Mann–Whitney test in case of non-normality. Qualitative variables were compared using the chi-squared test. The length of stay was studied by survival analysis according to the Kaplan–Meier method. The day of admission was considered as the starting point, while the end point was the date of discharge. Kaplan–Meier curves were compared by log-rank test.

Logistic regression models were used with the aim of exploring associations with AKI. We added in the univariate logistic regression analyses the parameters which resulted as associated (*p* < 0.05) to AKI after comparison of characteristics of the patients with and without AKI (Table 1). These parameters were grouped in anamnestic, clinical, and biochemical prognostic factors for AKI in order to give useful information and possible red flags for each of the steps of usual evaluation of patients. Among the anamnestic factors, duration of symptoms before hospitalization and birth weight (linear), and preterm birth (yes/no) were included. Among clinical factors, signs of respiratory distress, RR > 2 SDS, need of oxygen therapy, presence of fever, refill > 2 s, and the presence of severe pneumonia (yes/no) were included. Among biochemical factors, C-reactive protein (CRP) levels (linear) and hematocrit < 2 SDS (yes/no) were included. In the univariate analysis we did not include the (i) duration of oxygen therapy and length of stay, because these are only *a posteriori* available data; (ii) need of high flow oxygen therapy and of intubation because only one patient belonged to both groups, and (iii) need of 0.9% NaCl bolus because only the 4 patients with refill > 2 s underwent 0.9% NaCl bolus. The univariate analyses were performed to identify candidate variables to include in the multivariate analyses.

We included in the multivariate analyses only the variables with significant *P* < 0.05. We considered significant at multivariate analysis only the variables with significant p after Bonferroni correction. The significant p values after Bonferroni correction were < 0.016 for anamnestic prognostic factors, < 0.01 for clinical prognostic factors, and < 0.025 for biochemical prognostic factors. We included in the final multivariate analysis only the variables significant at multivariate analysis after Bonferroni correction. At the final multivariate analysis, the significant P cut-off after Bonferroni correction was 0.012.

Stat-Graph XVII software for Windows was used for all statistical analyses with the exception of logistic regression models made with SPSS 25 software for Windows.

## Results

One hundred and eighty-six patients (48.4% of male gender) aged 2.6 ± 2.4 years (range: 1 month–12.5 years; prevalence of children < 3 years of age: 69%) were enrolled (Fig. 1). All the patients presented X-ray confirmation of pneumonia. Eighty-nine (15 with empyema) (47.8%) patients had lobar pneumonia, 77 bronchopneumonia (41.4%), and 20 (10.8%) interstitial pneumonia. Fourteen out of 89 patients (15.7%) with lobar pneumonia, 20 out of 77 (26.0%) with bronchopneumonia, and 4 out of 20 (20%) with interstitial pneumonia presented AKI (*p* = 0.26).

Among the 15 patients with empyema, 4 underwent pleural drainage because of large effusions. Patients with empyema undergoing pleural drainage presented longer length of stay compared with patients with empyema not undergoing drainage (11.0 ± 2.8 vs. 7.4 ± 2.9; *p* = 0.05).

The general characteristics of the enrolled patients are shown in Table 1. None had previously known nephro-urological diseases.

All the patients needing intravenous rehydration underwent normal saline infusion.

Out of 186 patients, AKI was found in 38 (20.4%). In more detail, AKI was found in 20 out of 37 patients (54.0%) with severe and in 18 out of 149 (12.1%) with non-severe pneumonia (*p* < 0.001). No patient required hemodialysis nor reached AKI stage 3, 5 out of 38 (13.2%) reached AKI stage 2, and 33 (86.8%) reached AKI stage 1.

Among the 38 patients with AKI, 32 (84.2%) presented maximum AKI stage at hospitalization, 4 (10.5%) at 2 days, and 2 (5.3%) at 3 days.

**Fig. 2:**
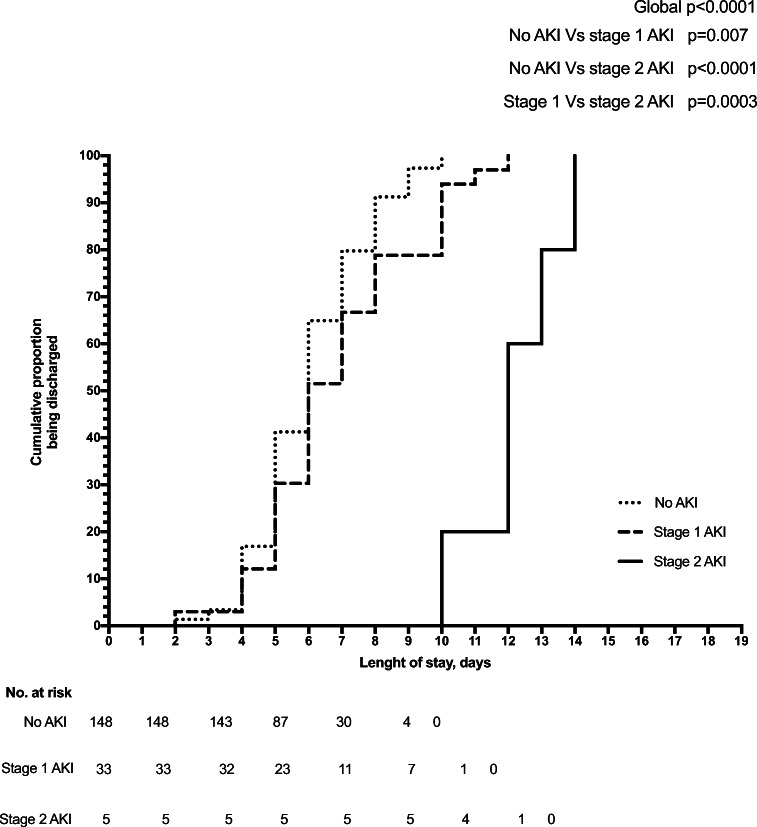
Length of stay evaluated by Kaplan-Meier analysis. The cumulative proportion of discharge of patients without AKI was 1.3% at 2 days, 3.4% at 3, 16.9% at 4, 41.2% at 5, 64.9% at 6, 79.7% at 7, 91.2% at 8, 97.3% at 9, and 100% at 10 days. For the patients with stage 1 AKI, the cumulative proportion of discharge was 3.0% at 2 days, 12.1% at 4, 30.3% at 5, 51.5% at 6, 66.7% at 7 and 78.8% at 8, 93.9% at 10, 97.0% at 11, and 100% at 12 days. For the patients with stage 2 AKI, the cumulative proportion of discharge was 0% until 9 days, 60% at 12, 80% at 13 and 100% at 14 days. Log-rank test comparing the three Kaplan–Meier curves showed a global *p* < 0.001 (no vs. stage 1 AKI, *p* = 0.007; stage 1 vs. stage 2 AKI, *p* = 0.0003; no vs. stage 2 AKI, *p* < 0.0001)

The mean length of stay was 6.0 ± 1.7, 6.9 ± 2.3, and 12.2 ± 1.5 days, for patients without AKI, with stage 1 AKI and stage 2 AKI (*p* < 0.001), respectively. These findings were also confirmed by the Kaplan–Meier analysis that showed the shortest time to discharge for patients without AKI, the longest time for patients with stage 2, and intermediate time for patients with stage 1 AKI (Fig. 2).

Patients with AKI presented lower birth weight, higher duration of symptoms before admission, duration of oxygen therapy, serum urea levels, CRP levels, and length of stay such as higher prevalence of preterm birth, signs of respiratory distress, RR > 2 SDS, need of oxygen therapy, need of high flow oxygen therapy, need of intubation, presence of fever, refill > 2 s, severe pneumonia, need of bolus, and hematocrit < 2 SDS, compared with patients without AKI (Table 1). No differences in the percentage of nephrotoxic drugs utilization were evident comparing patients with and without AKI.

At univariate logistic regression analyses, duration of symptoms before hospitalization, birth weight, preterm birth, signs of respiratory distress, RR > 2 SDS, need of oxygen therapy, presence of fever, refill time > 2 s, presence of severe pneumonia, CRP levels, and hematocrit < 2 SDS resulted significant and then were included in the multivariate analyses (Table 2). At multivariate logistic regression analyses, the only significant prognostic factors were duration of symptoms before hospitalization among the anamnestic factors, presence of fever and severe pneumonia among the clinical factors, and CRP levels among the biochemical factors (Table 2). A final multivariate analysis including all these parameters showed that only duration of symptoms before hospitalization, severe pneumonia, and CRP levels were independent predictors of AKI (Table 2).

**Table 2:** Exploratory analysis of prognostic factors potentially associated with AKI

	**Univariate analysis**^**d**^			**Multivariate analysis**^**d**^			**Final multivariate analysis**^**d**^		
**Anamnestic prognostic factors**	**OR**	**95% CI**	**p**	**OR**	**95% CI**	**p**	**OR**	**95% CI**	**p**
Duration of symptoms before hospitalization^a^, days	1.2	1.10/1.30	0.001	1.2	1.01/1.30	0.002	1.2	1.09/1.43	0.001
Birth weight^b^, kg	1.9	1.02/3.40	0.04	1.7	0.80/3.70	0.17	-	-	-
Preterm birth	2.4	1.02/5.80	0.04	1.5	0.50/4.80	0.48	-	-	-
**Clinical prognostic factors**	**OR**	**95% CI**	**P**	**OR**	**95% CI**	**p**	**OR**	**95% CI**	**p**
Signs of respiratory distress	2.4	1.10/5.10	0.02	3.0	0.68/13.56	0.15	-	-	-
Respiratory rate >2SDS	3.0	1.03/8.90	0.04	2.7	0.75/9.80	0.13	-	-	-
Need of oxygen therapy	3.9	1.50/10.20	0.006	4.9	0.66/37.7	0.12	-	-	-
Presence of fever	3.1	1.40/6.90	0.005	13.1	2.1/83.5	0.006	1.9	0.72/5.09	0.19
Refill >2 seconds	6.3	1.01/38.80	0.049	2.0	0.05/82.90	0.71	-	-	-
Severe Pneumonia	8.6	3.8/19.3	<0.001	46.5	3.4/642.8	0.004	11.9	4.30/33.30	<0.001
**Biochemical prognostic factors**	**OR**	**95% CI**	**p**	**OR**	**95% CI**	**p**	**OR**	**95% CI**	**p**
C-reactive protein^c^, mg/dL	1.1	1.03/1.22	0.001	1.1	1.05/1.22	0.001	1.1	1.04/1.23	0.004
Hematocrit <2SDS	2.2	1.03/4.50	0.04	1.4	0.59/2.9	0.49	-	-	-

Goodness-of-fit of the models: for the multivariate analysis of anamnestic, clinical and biochemical prognostic factors, the Nagelkerke’s R^2^ and the p of the Hosmer and Lemeshow test are 0.15 and 0.55, 0.53 and 0.91, and 0.13 and 0.1, respectively. For the final multivariate analysis, the Nagelkerke’s R^2^ is 0.43 and the p of the Hosmer and Lemeshow test is 0.98.

^a^1 day increase in symptoms duration

^b^1 kg increase in birth weight

^c^1 mg/dL increase in C-reactive protein levels.

^d^We included in the multivariate analysis only the variables with *p* < 0.05 at univariate analysis. We considered significant at multivariate analysis only the variables with significant p after Bonferroni correction. The significant p values after Bonferroni correction were < 0.016 for anamnestic risk factors, < 0.01 for clinical risk factors, and <0.025 for biochemical risk factors. We included in the final multivariate analysis only the variables significant at multivariate analysis after Bonferroni correction. At the final multivariate analysis, the significant p cut-off after Bonferroni correction was 0.012

*Abbreviations:* *AKI* acute kidney injury, *SDS* standard deviation score

## Discussion

It is reported that one half of the adults hospitalized for CAP develop sepsis, with non-pulmonary organ dysfunction developing in more than one third [20]. The AKI prevalence in adults with CAP ranges from 4.3 to 34% [2–5].

In children with CAP, we found AKI prevalence of 20.4%. AKI was mostly of mild degree in our series. Similarly to findings in adults [2–5], we found higher prevalence of AKI among children with severe compared with non-severe pneumonia.

In adults, factors predicting development of AKI include severity of pneumonia, elevated CRP, and previous need of angiotensin-converting enzyme inhibitors or angiotensin-II-receptor blockers [3]. Moreover, higher biomarkers of inflammation (interleukin-6, -10, and tumor necrosis factor) concentrations in patients with CAP developing AKI compared with those not developing AKI were reported [2]. We found that duration of symptoms before hospitalization, severity of pneumonia, and serum CRP levels were significant and independent predictors for AKI in children with CAP. On the other hand, clinical signs that usually are associated with dehydration, such as refill > 2 s, HR > 2 SDS, and hematocrit > 2 SDS, were not significant predictors. Similarly, the use of nephrotoxic drugs did not play a relevant role in the development of AKI (Table 1).

Therefore, differently from the setting of acute gastroenteritis in children where prognostic factors for AKI are strictly linked to dehydration [21], in children with CAP the pneumonia severity and the degree of systemic inflammatory response appear to be particularly important in AKI development.

In our series, the length of stay was longer for patients with stage 2 AKI, intermediate for patients with stage 1 AKI and shorter for those without AKI (Fig. 2). Similar data are reported in adults with CAP [2, 3]. In our population, however, the higher length of stay of patients with stage 1 AKI (6.9 days on average) compared with those without AKI (6 days on average) was not relevant from a practical point of view. Actually, mild forms of AKI easily and quickly resolved. Therefore, in the short term, stage 1 AKI during a CAP episode does not carry a major risk of acute complications. From a long-term perspective, however, the identification of mild forms of AKI could be relevant to schedule a proper follow-up for these patients with periodical serum creatinine, proteinuria, and blood pressure evaluation [7], in order to monitor the risk of later CKD [6].

Pediatricians should be aware that repeated AKI episodes can increase the risk of CKD in adulthood [22], and it could be useful to alert parents of these patients to avoid—as far as possible—further AKI episodes in their children.

Of note, none of the patients having shown AKI during CAP in our retrospective analysis had undergone nephrological follow-up nor kidney ultrasound for this episode, confirming our impression that often AKI could be overlooked.

Our study has several limitations: (i) the retrospective design; (ii) lack of a real basal serum creatinine level (before hospitalization) and very limited data about its levels at discharge, which did not allow us to retrospectively evaluate the rate of serum creatinine normalization; (iii) unavailability to adopt the KDIGO urinary output criteria with possible underestimation of AKI prevalence; and (iv) not all patients underwent second creatinine determination and, in those undergoing it, the second determination was made with irregular interval time, on the basis of clinical judgement. One bias could be that some of the 55 patients without a second creatinine measurement could have developed AKI later during hospitalization. Considering that among the 38 patients with AKI, we found AKI at the second creatinine measurement in 6 (15.8%), it is possible that we missed AKI in 15.8% of the 55 (*n* = 8.7) patients missing the second creatinine determination. However, supposing that the patients not having undergone second blood sample collection were those with better clinical conditions, it is possible that the number of missed AKI is not relevant.

In conclusion, we found that about one fifth of children hospitalized for CAP may suffer from mostly mild AKI with a longer stay for those with more severe AKI. The AKI prevalence was higher in children with severe compared with non-severe pneumonia. Main prognostic factors for AKI are represented by duration of symptoms before hospitalization, severity of pneumonia, and serum CRP levels. Attention should be paid to the kidney health of these patients to avoid further factors predisposing to AKI, such as dehydration [21]. On the basis of our pilot retrospective observation, future prospective studies on AKI in children with CAP could be performed in order to improve the kidney care of children with CAP.

## Supplementary Information

ESM 1 (PPTX 362 kb).

## Data Availability

The dataset generated during and/or analyzed during the current study are available from the corresponding author on reasonable request.
