# Supplementary material for: Acute kidney injury in children hospitalized for community acquired pneumonia
Source: Pediatr Nephrol. 2021 Mar 20;36(9):2883–90. doi: 10.1007/s00467-021-05022-x (PMC8370910; doi:10.1007/s00467-021-05022-x)
Supplement: Supplementary file 1 — (PPTX 362 kb). [file 467_2021_5022_MOESM1_ESM.pptx]

## Slide 1
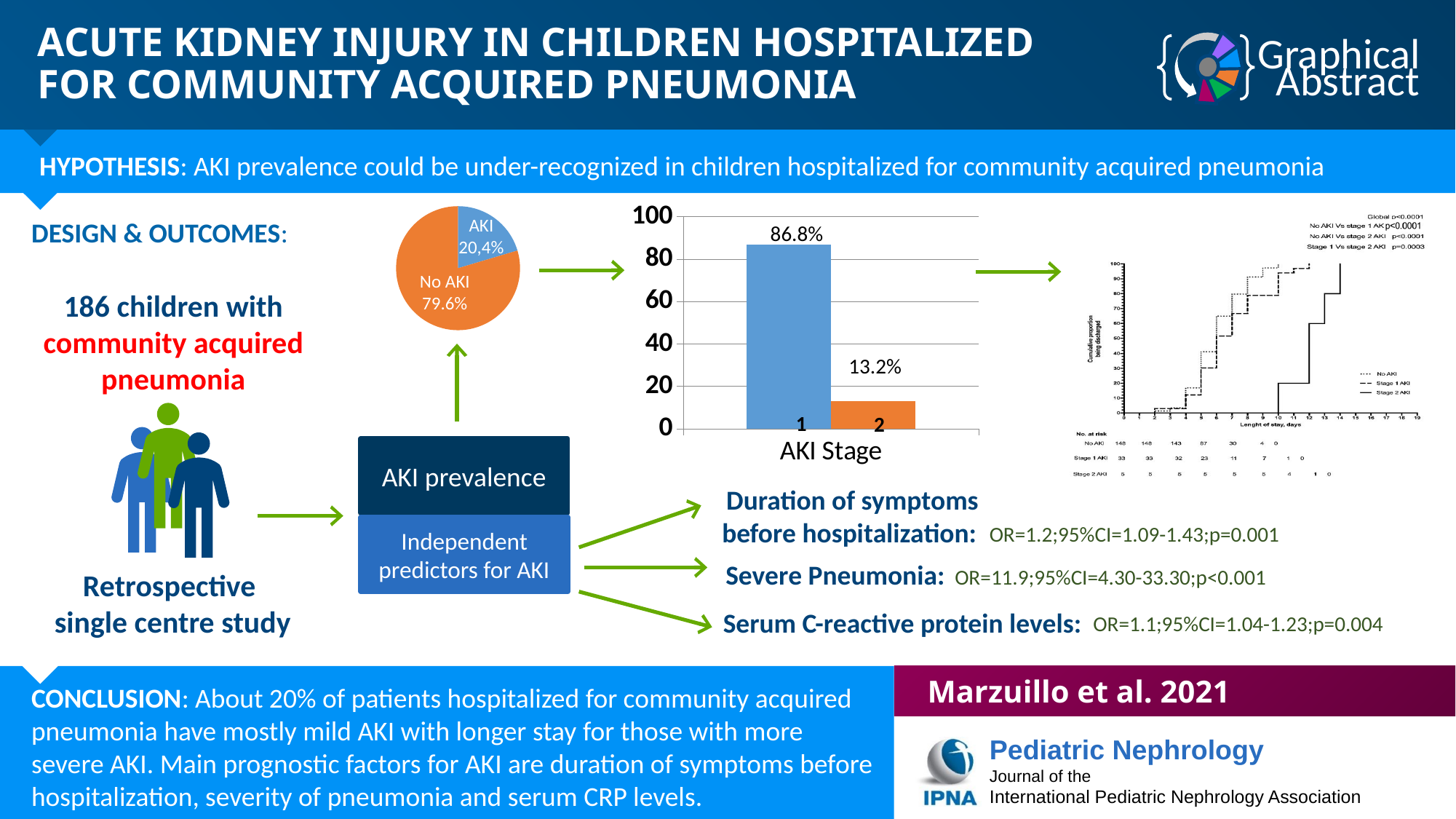

Acute Kidney Injury in children hospitalized for community acquired pneumonia
HYPOTHESIS: AKI prevalence could be under-recognized in children hospitalized for community acquired pneumonia
### Chart
| Category | AKI prevalence |
|---|---|
| AKI | 20.4 |
| No AKI | 79.6 |AKI
20,4%
No AKI
79.6%
### Chart
| Category | Mild | Severe |
|---|---|---|
| AKI Stage | 86.8 | 13.2 |DESIGN & OUTCOMES:
### Chart
| Category |
|---|
86.8%
186 children with community acquired pneumonia
13.2%
 1
 2
AKI prevalence
Independent predictors for AKI
Duration of symptoms before hospitalization:
OR=1.2;95%CI=1.09-1.43;p=0.001
Severe Pneumonia:
OR=11.9;95%CI=4.30-33.30;p<0.001
Retrospective
single centre study
Serum C-reactive protein levels:
OR=1.1;95%CI=1.04-1.23;p=0.004
Marzuillo et al. 2021
CONCLUSION: About 20% of patients hospitalized for community acquired pneumonia have mostly mild AKI with longer stay for those with more severe AKI. Main prognostic factors for AKI are duration of symptoms before hospitalization, severity of pneumonia and serum CRP levels.
